# Submucosal Intraglossal Fish Bone Extraction: A Case for the Multidisciplinary Team

**DOI:** 10.7759/cureus.20263

**Published:** 2021-12-08

**Authors:** Benjamin M Olley, Yinan Zhu, Leyla Ozbek, Thomas Ringrose, Catherine Lau

**Affiliations:** 1 Head and Neck Surgery, University College Hospital, London, GBR; 2 Oral and Maxillofacial Surgery, University College Hospital, London, GBR

**Keywords:** posterior tongue, oral and maxillofacial pathology, ultrasound guided intervention, fish bone perforation, foreign body removal

## Abstract

Most impacted fish bones in the aerodigestive tract are easily removed or managed in the emergency department. Occasionally, they present as a diagnostic and surgical challenge. We present a case of a submucosal intraglossal fish bone extraction in a 38-year-old male who presented with localized pain in his tongue. This case highlights several key factors contributing to the successful outcome, including multidisciplinary input from anaesthesiology, radiology, and the oral and maxillofacial surgical team. The use of a pre-operative computed tomography (CT) scan, nasal intubation, and intra-operative ultrasound scan potentially minimised the risk of associated complications.

## Introduction

Upon ingestion, fish bones can perforate the mucosa of any section of the digestive tract and become impacted. In adults, these are most commonly impacted in the oropharynx, oral cavity, and oesophagus, in this order [1]. Clinical presentation can vary greatly, from localized pain and sensation of a foreign body to neck swelling and pyrexia [1]. Most patients, however, are discharged after primary management in the emergency department [2].

Here, we present a rare case of a submucosal intraglossal fish bone impaction that required multidisciplinary input prior to a successful extraction in the operating theatre with the help of intra-oral ultrasound guidance. Only a few complicated cases like these have been reported in the literature [3-5], with varying management strategies. The focus of this case report will be on the investigation and management considerations of such a presentation to a well-resourced tertiary Oral and Maxillofacial Surgery (OMFS) Head and Neck unit.

## Case presentation

A 38-year-old male presented with localized pain in the left lateral tongue and foreign body sensation persisting since a fish and rice meal three days previously. He felt a penetrating sensation during his meal and thought he was able to remove the fish bone from his tongue but the pain did not improve. The patient was otherwise systemically well and able to tolerate a normal diet with some discomfort. He has no significant past medical history, no medications, and no allergies. Upon palpation of the tongue, there was a tender indurated focal point in the posterior left lateral aspect near the region of the circumvallate papillae. No foreign body was visualised with further inspection of the oral cavity, oropharynx, and on flexible nasendoscopy. Lateral neck x-rays taken were unremarkable.

**Figure 1:**
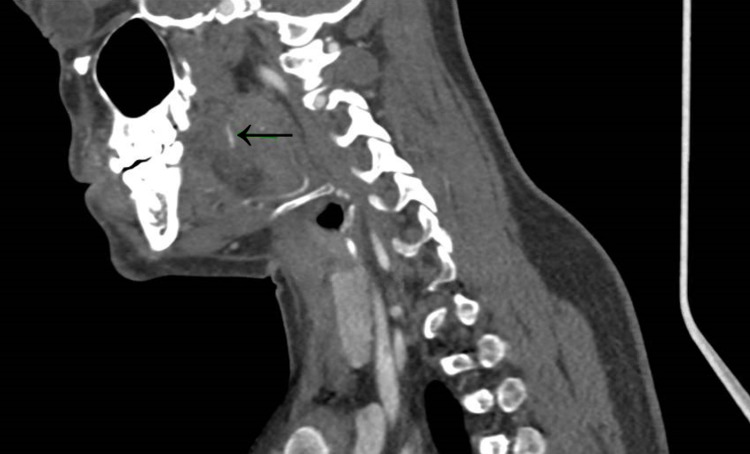
Sagittal contrast-enhanced CT of neck: suspected intraglossal fish bone visible in caudocranial orientation Black arrow points to the suspected fish bone

**Figure 2:**
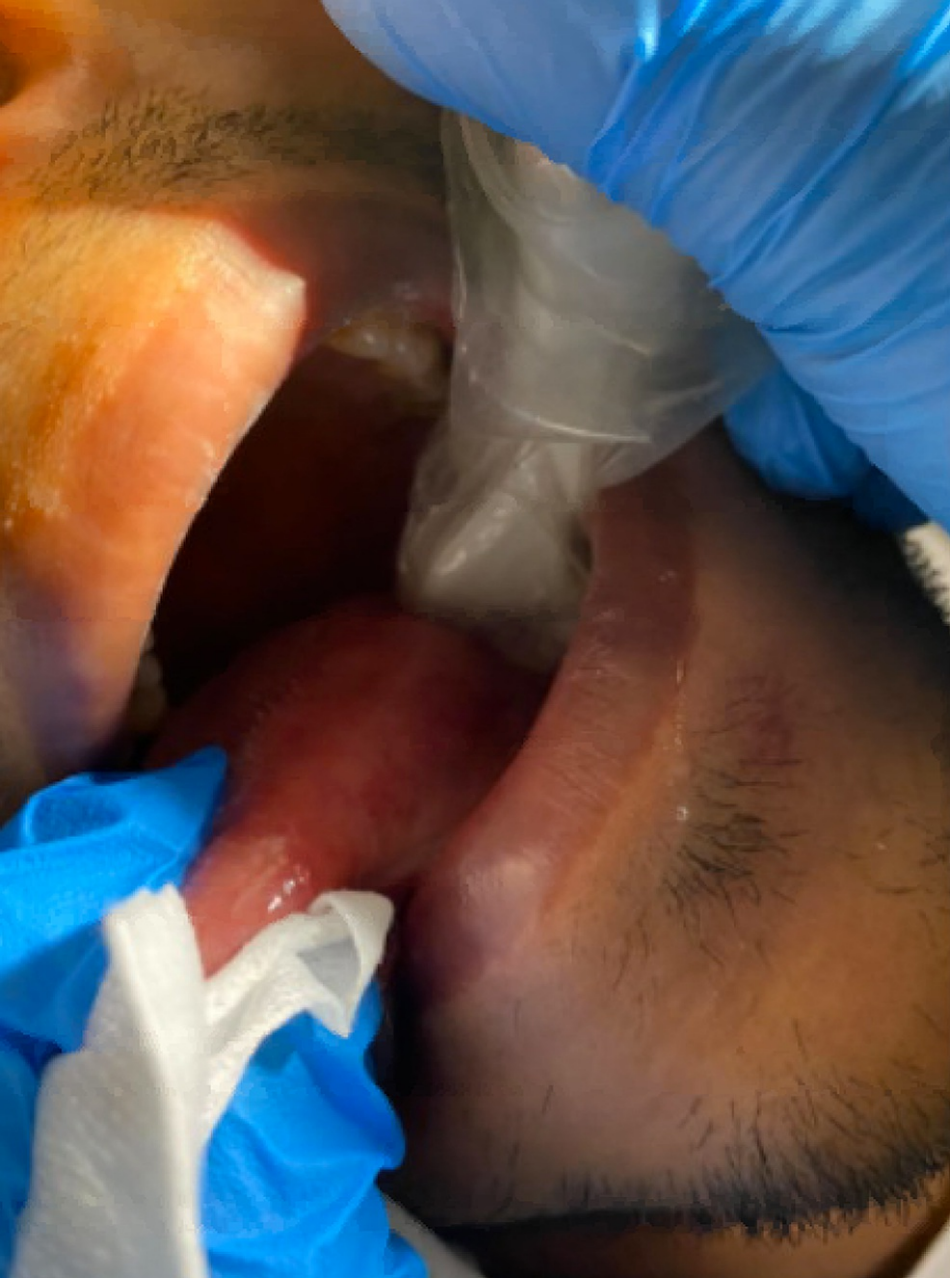
Intraoral ultrasound probe on the left lateral tongue

**Figure 3:**
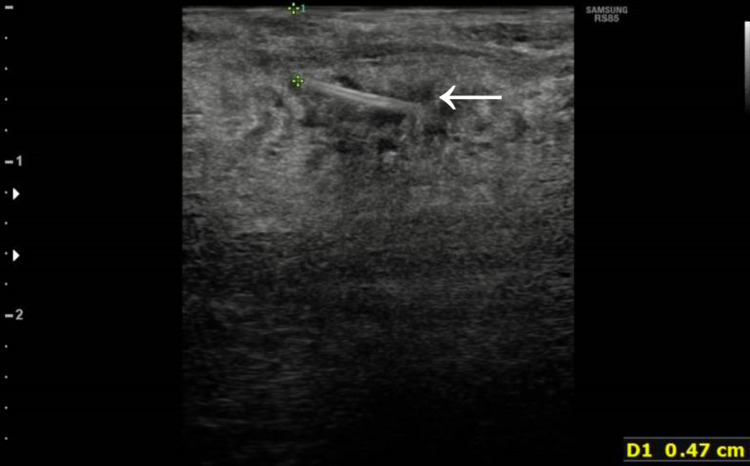
Ultrasound scan still image of intraglossal foreign body (<1mm diameter, 9mm long, 4.7mm deep at the most superficial aspect) White arrow points to the suspected fish bone

Contrast-enhanced computed tomography (CT) of the neck was performed, which demonstrated a 9mm linear hyperdensity in the left superior aspect of the tongue (Figure 1). However, as this was a contrast study it could have also represented an unusual blood vessel. As such, an intra-oral ultrasound was performed with the tongue protruding and the probe against the posterior left lateral tongue (Figure 2). This confirmed the presence of a foreign body <1mm diameter lying in the anteroposterior position with its most superficial aspect 4.7mm deep to the mucosal surface (see Figure 3). 

**Figure 4:**
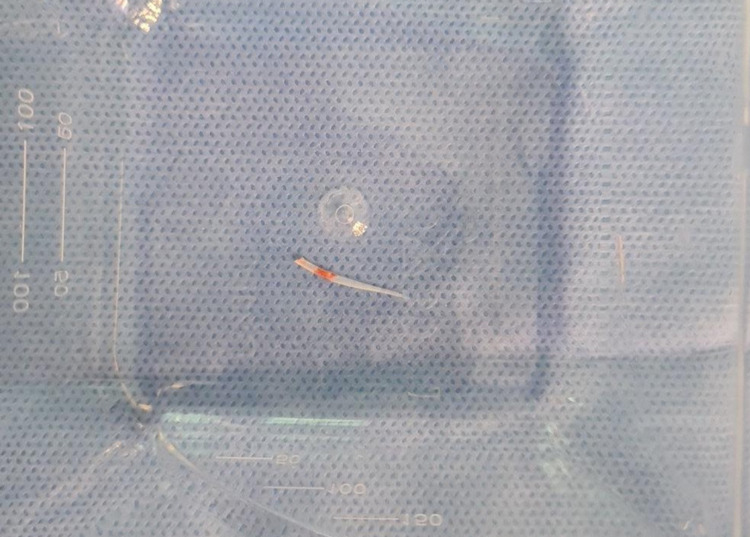
Fish bone removed

Following a multi-disciplinary discussion between the radiologist and OMFS surgeon, the patient consented to the removal of the foreign body under general anaesthetic with ultrasound guidance. The patient was positioned supine following awake fibre-optic nasal intubation. The tongue was retracted with a suture into a protruded rightward position. The radiologist marked the position of the foreign body under live ultrasound guidance and the OMFS surgeon made a small mucosal incision. The radiologist then manipulated the tip of an artery clip to grasp the foreign body. At this stage, the foreign body did not move freely. The incision was then extended onto the clip until the foreign body was removed in its entirety (Figure 4). Haemostasis was achieved and the mucosa was subsequently closed with absorbable sutures. The patient was discharged the following day and recovered in the community without complications.

## Discussion

Our patient has had a successful uncomplicated extraction. However, the theoretical simplicity of an intraglossal impaction of fish bone could potentially turn into a surgical nightmare as a thin well-buried bone could be tricky to identify within the adjacent inflamed tongue muscle [6]. If not cautiously and timely managed, this can lead to serious complications including an intraglossal abscess, airway compromise, and in one case, the need for partial glossectomy due to difficulty locating the foreign body [3]. As such, if there is any doubt in the emergency department upon the initial assessment, we recommend a direct referral to OMFS or a head and neck speciality for advice.

Removal of the foreign body under general anaesthetic was necessary in this case. Local anaesthetic was not considered to minimize patient movement and discomfort due to the location of the fish bone. In addition, sensation to the posterior third of the tongue is supplied by the glossopharyngeal nerve and this nerve block carries a risk of injury to surrounding great vessels of the neck [7]. The choice of the general anaesthetic approach is also critical. Intubating orally with a laryngoscope risked further impacting the fish bone into the tongue. This could have made exploration more difficult and traumatic. Awake nasal fibre-optic intubation mitigates this risk. It does require advanced airway equipment and skill, but this should be available in hospitals providing head and neck services [8].

Pre-operative imaging can not only help to identify a submucosal fish bone but is also important in delineating its approximate anatomical location and thus reduce the trauma of exploration. Although convenient, a thorough oropharyngeal examination and plain x-ray lateral neck films are less sensitive and consistent than a CT neck scan in visualising fish bones within soft tissues [9], although we were unable to find any evidence supporting or against contrast administration. Intra-operatively, It should be noted that the tongue remains in the oral cavity during a CT scan and is protruded during exploration, hence the orientation of the foreign body may not lie exactly as expected.

Intra-oral ultrasound can clearly delineate fish bones (Figure 3). Ultrasound is a non-irradiating operator-dependent imaging modality and experience in its use in the oral cavity is uncommon [10]. Intra-operatively, an experienced head and neck radiologist was able to direct the surgeon accurately to the site of the fish bone and it was removed with minimal trauma. This may have been less successful with non-specialist equipment (e.g. larger probe) or in patients with more limited mouth opening, reduced tongue mobility, or a more posteriorly located fish bone. Intra-operative CT scanning is also a viable option and has been used successfully in one case [5], but this does come with additional risk from radiation exposure [11]. CT scanners are widely available but their usage during operative procedures is often limited to interventional suites in highly specialist centres. Ultimately, the decision on which intra-operative modality to use should depend on the speciality, equipment and patient factors, tailored to the case.

## Conclusions

While most oropharyngeal fish bone impactions are uncomplicated and successfully managed in the emergency department, if it is suspected in an unusual location, e.g. intraglossal, the patient should be discussed with OMFS or a head and neck speciality before the first attempt at extraction is made. Intraglossal fish bone impactions can have life-threatening complications and additional imaging should also be performed to confirm and delineate the foreign body. In cases requiring removal under general anaesthesia, nasal intubation provides better access and reduces the risk of fish bone migration. Ultrasound is an excellent choice of intra-operative imaging if local expertise, equipment, and intra-oral access allows.
